# Combination of Erythromycin and Curcumin Alleviates *Staphylococcus aureus* Induced Osteomyelitis in Rats

**DOI:** 10.3389/fcimb.2017.00379

**Published:** 2017-08-24

**Authors:** Zubin Zhou, Chenhao Pan, Ye Lu, Youshui Gao, Wei Liu, Peipei Yin, Xiaowei Yu

**Affiliations:** Department of Orthopaedic Surgery, Shanghai Jiao Tong University Affiliated Sixth People's Hospital Shanghai, China

**Keywords:** *Staphylococcus aureus*, osteomyelitis, erythromycin, curcumin, inflammation

## Abstract

Osteomyelitis is commonly caused by *Staphylococcus aureus*. Both erythromycin and curcumin can suppress *S. aureus* growth, but their roles in osteomyelitis are barely studied. We aim to explore the activities of erythromycin and curcumin against chronical osteomyelitis induced by methicillin-resistant *S. aureus* (MRSA). Chronicle implant-induced osteomyelitis was established by MRSA infection in male Wistar rats. Four weeks after bacterial inoculation, rats received no treatment, erythromycin monotherapy, curcumin monotherapy, or erythromycin plus curcumin twice daily for 2 weeks. Bacterial levels, bone infection status, inflammatory signals and side effects were evaluated. Rats tolerated all treatments well, with no death or side effects such as, diarrhea and weight loss. Two days after treatment completion, erythromycin monotherapy did not suppress bacterial growth and had no effect in bone infection, although it reduced serum pro-inflammatory cytokines tumor necrosis factor (TNF)-α and interleukin (IL)-6. Curcumin monotherapy slightly suppressed bacterial growth, alleviated bone infection and reduced TNF-α and IL-6. Erythromycin and curcumin combined treatment markedly suppressed bacterial growth, substantially alleviated bone infection and reduced TNF-α and IL-6. Combination of erythromycin and curcumin lead a much stronger efficiency against MRSA induced osteomyelitis in rats than monotherapy. Our study suggests that erythromycin and curcumin could be a new combination for treating MRSA induced osteomyelitis.

## Introduction

*Staphylococcus aureus* is the most common pathological microorganism that causes osteomyelitis (Lew and Waldvogel, 2004). Effective treatment for osteomyelitis is still lacking due to the formation of biofilm rendering it difficult for sufficient antibiotics to reach the local infection area as well as the development of resistance to antibiotic treatment. Although some drugs can temporarily suppress methicillin-resistant *S. aureus* (MRSA) induced osteomyelitis in animal models, severe side effects are observed, such as, diarrhea, malnutrition, and sometimes loss of research animals (Vergidis et al., 2015). Development of new safe and effective therapeutic strategies is highly demanded to overcome drug-resistant bacterial strains and adverse side effects. One such strategy is combination of different antibiotics or with plant derived natural products (Coutinho et al., 2009; Bezerra Dos Santos et al., 2015).

Erythromycin is broadly used to treat infections caused by various bacteria, including *S. aureus* (Jelic and Antolovic, 2016). The first use of erythromycin in clinical setting dated back to the early 1950s. The key mechanism for antibiotics of the macrolide family including erythromycin in suppressing bacterial growth is to inhibit the production of proteins crucial for bacterial function. In addition to direct targeting against bacteria, erythromycin also alleviates bacterial infection symptoms by suppressing inflammation (Cervin, 2001; Amsden, 2005). Erythromycin has been shown to reduce the production of pro-inflammatory cytokines in various scenarios (Miyajima et al., 1999; Desaki et al., 2004). While *S. aureus* frequently evolves to acquire resistance to antibiotic treatment, combination of erythromycin with other antibiotics or herbal medicines seems to be able to overcome this difficulty and results in a synergistic effect in suppressing bacterial growth (Bezerra Dos Santos et al., 2015). Interestingly, although the efficacy of erythromycin has been reported in various scenarios, its role in treating MRSA induced osteomyelitis is not well documented.

Some naturally occurring flavonoids have been shown to possess antibacterial functions against MRSA and can work synergistically with antibiotics such as, erythromycin to suppress bacterial growth (Chan et al., 2013). Curcumin, a major constituent of turmeric commonly used as a food spice, is a natural polyphenolic flavonoid isolated from the rhizome of the plant Curcuma longa Linné. Curcumin has been shown to exhibit various biological functions, including anti-oxidant, anti-inflammatory, anti-tumorigenic activities (Wang et al., 2009; Hussain et al., 2017; Zhao et al., 2017). It down-regulates pro-inflammatory signaling pathways, including transcription factors, cytokines, and other components that promote inflammation (Shehzad et al., 2013). It also possesses anti-microbial functions, including activity against *S. aureus* (Mishra et al., 2005; Rai et al., 2008; Tyagi et al., 2015). In addition, curcumin works synergistically with other antibiotics including oxacillin, ampicillin, ciprofloxacin, and norfloxacin against MRSA and significantly reduces the minimal inhibitory concentrations (MICs) of these antibiotics (Mun et al., 2013; Teow and Ali, 2015). Another *in vitro* study suggests that curcumin can reverse methicillin resistance in *S. aureus* (Mun et al., 2014). These observations lead us to hypothesize that curcumin may also be effective in suppressing MRSA growth in osteomyelitis.

In the current study, we explored the efficacies and side effects of erythromycin monotherapy, curcumin monotherapy, and erythromycin and curcumin combined therapy in a rat implant-induced osteomyelitis model caused by MRSA infection.

## Materials and methods

### Bacteria

*S. aureus* ATCC 43300 (a standard MRSA isolate; ATCC) was used to establish chronic osteomyelitis in rats.

### Rat osteomyelitis model

All animal experiments were conducted in accordance with guidelines set by USA National Institutes of Health and standard protocols on materials with biosafety issue set by Shanghai Jiao Tong University Affiliated Sixth People's Hospital's Institutional Animal Care and Use Committee. This protocol of the animal study was also approved by Shanghai Jiao Tong University Affiliated Sixth People's Hospital's Institutional Animal Care and Use Committee (20160304). Adult male Wistar rats weighing 260 g to 340 g were purchased from SLAC Company and housed in Shanghai Jiao Tong University Affiliated Sixth People's Hospital rat facility under standard environment with free access to water and food. The procedures of establishing implant related osteomyelitis were adapted from previous studies (Vergidis et al., 2015; Guzel et al., 2016). Briefly, experimental rats were anesthetized by intraperitoneal injection of weight adjusted ketamine (60 mg/kg body weight) and xylazine (6 mg/kg body weight). The left tibia was exposed after a small incision of the skin of the left hind limb. A hole was drilled in the bone using a 1 mm diameter titanium burr to allow access to medullary cavity. 10 μL suspension containing 10^8^ colony-forming unit (CFU) of MRSA was injected into the medullary cavity of each exposed tibia. Following that, a 5 mm (length) ×1 mm (diameter) titanium Kirschner wire was inserted into the medullary cavity. Dental gypsum was used to cover the hole and the skin was sutured and treated with antiseptic solution. Subcutaneous administration of Buprenorphine was used for analgesia. Four weeks after surgery and bacteria inoculation, rats were randomly assigned to four groups, with 14 rats per group: control (no treatment), erythromycin monotherapy, curcumin monotherapy, and erythromycin plus curcumin. Both drugs were injected intraperitoneally every 12 h for 14 days. The dose for erythromycin (Sigma, MO, USA) is 20 mg/kg body weight and the dose for curcumin (Sigma, USA) is 50 mg/kg body weight. All rats were closely monitored for signs of adverse effects such as diarrhea and weight loss. 48 h after completion of drug treatment, rats from each study group were euthanized for MSRA culture and quantification, and histopathological evaluations.

### Microbiological assessment

Culture and quantification of bacteria from bone and wire were described previously (Vergidis et al., 2015). Briefly, the left tibia of each rat was aseptically removed. The bone were weighed and homogenized respectively. The homogenates were suspended in Trypticase soy broth, vortexed, diluted serially, plated onto sheep blood agar plates and cultured at 37°C. 48 h after incubation, MRSA colonies were quantified and the results were expressed as log_10_ CFU/g of bone and log_10_ CFU/cm^2^ of wire surface.

### Histopathological assessment

48 h after treatment, tibia from rats of each treatment group were fixed in formaldehyde and sectioned into 5 μm thick longitudinal sagittal sections. Bone slices were stained with hematoxylin and eosin (H&E) using standard procedures as described previously (Fischer et al., 2008). Four regions of interest (ROI) were assessed for bone infection, including proximal epi-/metaphysis and diaphysis, and distal epi-/metaphysis and diaphysis. Each ROI was assessed by four independent observers, with no access to the animal group codes. Scores were assigned based on the criteria as described in previous studies (Lucke et al., 2003; Guzel et al., 2016). The following four parameters were scored with 0 (absent) or 1 (present): 1. Abscess formation; 2. Sequestrum formation; 3. Enlargement of corticalis; 4. Destruction of corticalis. The remaining 5th parameter (general impression) was scored as 0 (absent), 1 (mild), or 2 (severe). The maximum score of one tibia including four ROIs and five parameters is 24.

### Elisa

Levels of tumor necrosis factor-alpha (TNF-α) and interleukin-6 (IL-6) in rat serum were detected by rat enzyme-linked immunosorbent assay (ELISA) kits (both purchased from R&D Biosystem, Minneapolis, MN, USA) according to manufacturer's instruction. Results were represented as pg/mL of serum. Serum from rats of each group was collected 48 h after treatment.

### Statistical analysis

Data were analyzed by SPSS 21.0 (SPSS Inc., Chicago, IL, USA) and presented as mean ± *SD*. The difference between two groups was determined by one-way ANOVA analysis followed by a Tukey's post hoc test. The difference was regarded statistical significant when *p* value < 0.05.

## Results

### Adverse effects of erythromycin and/or curcumin treatments in rats with osteomyelitis

**Figure 1:**
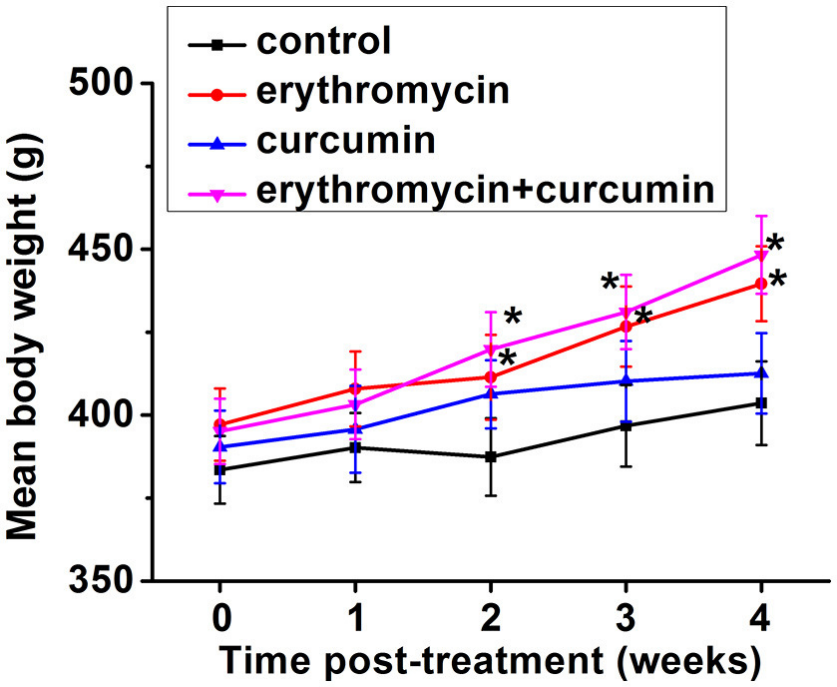
Mean body weight in each experimental group at indicated time points post treatment. Results were presented as mean ± *SD* ^*^p < 0.05 vs. control.

We closely monitored all the rats undergoing different treatments, including those in the control group. We found that the rats tolerated all the treatments well and all rats survived the whole study duration. No rats developed diarrhea or weight loss. In fact we observed a significant increase of body weight from 2 weeks post treatment when rats were treated with erythromycin alone or a combination of erythromycin and curcumin, compared to that of control group (Figure 1). Curcumin treatment alone did not significantly alter rat body weight.

### Combination of erythromycin and curcumin strongly suppressed MRSA growth

**Figure 2:**
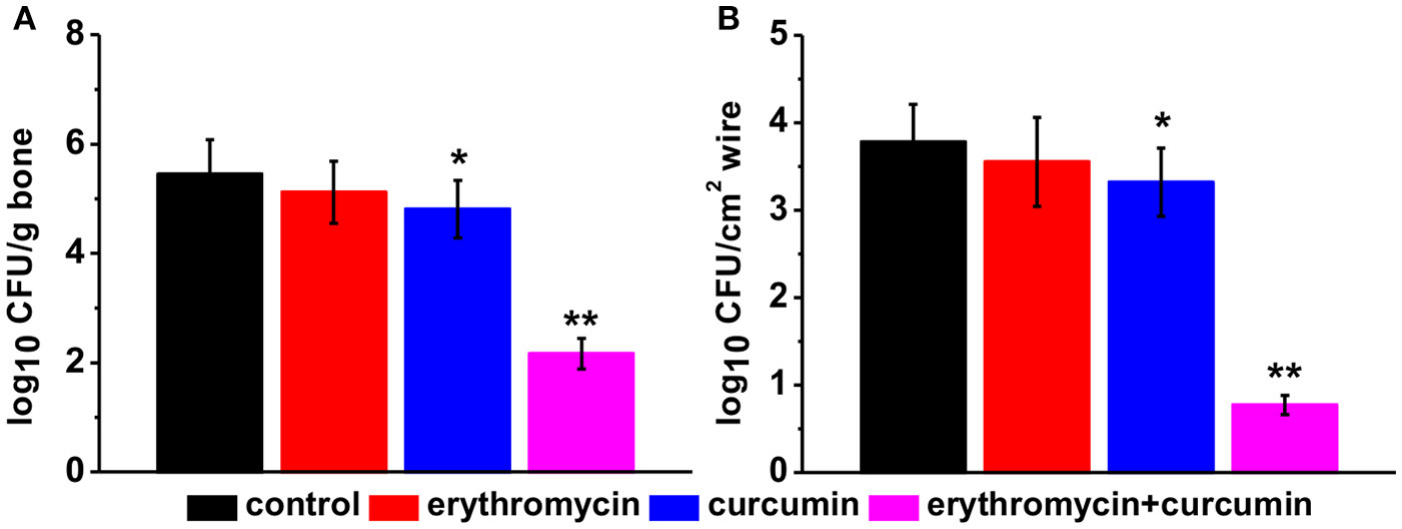
Bacterial levels in each experimental group 48 h post treatment, quantified in bone **(A)** and wire **(B)** cultures expressed as log_10_ colony forming units (CFU) per tissue (g) and per wire surface (cm^2^), respectively. Results were presented as mean ± *SD* ^*^p < 0.05 and ^**^p < 0.01 vs. control.

We evaluated bacterial growth in rats from all four groups 48 h after treatment. We found that treatment of rats with erythromycin alone had no effect on bacterial levels compared to that of control rat. Curcumin treatment alone slightly but significantly suppressed bacterial growth. Erythromycin and curcumin double treatment drastically reduced bacterial levels both in the bone (Figure 2A) and wire (Figure 2B). These results suggest that erythromycin and curcumin had a strong effect on suppressing MRSA growth.

### Reduced bone infection as a result of erythromycin and curcumin double treatment

**Figure 3:**
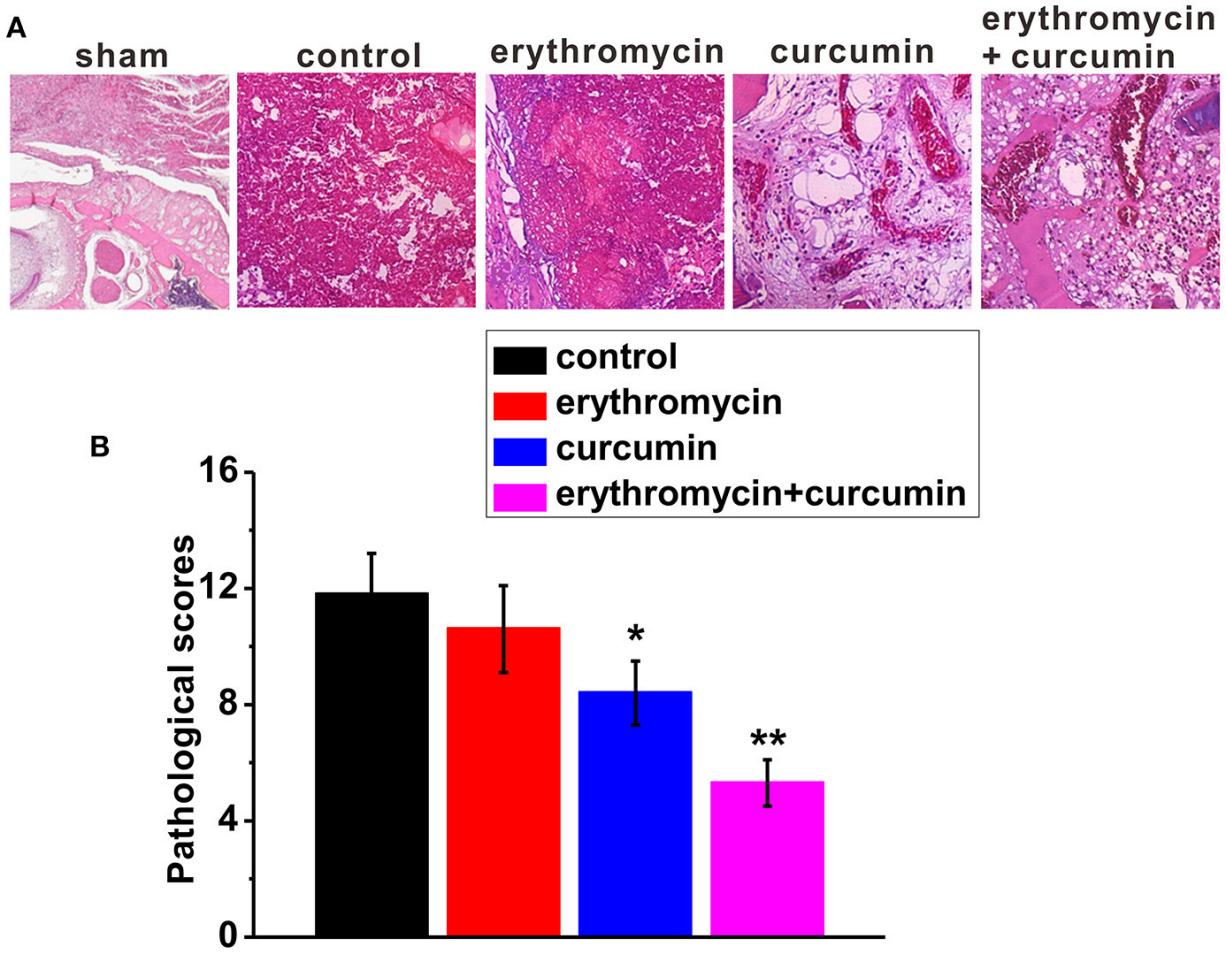
Histological examination in the tibiae of experimental rats. Representative images of H&E staining **(A)** and histological scores **(B)** in each experimental group. Note that the representative image of sham was from a healthy bone. Results were presented as mean ± *SD* ^*^p < 0.05 and ^**^p < 0.01 vs. control.

Forty-eight hour after treatment of rats with osteomyelitis, we examined bone tissue lesions using H&E staining (Figure 3A). We found that control rats showed severe signs of infection with a high average histopathological score (Figures 3A, B). Erythromycin treatment alone did not alleviate the status of osteomyelitis and the histopathological score was similar to that of control rats. Curcumin treatment alone slightly reduced bone infection with a significantly lower histopathological score. Importantly, combination of erythromycin and curcumin substantially suppressed bone lesions and decreased the histopathological score.

### Reduced pro-inflammatory cytokine levels in erythromycin and curcumin double treated rats

**Figure 4:**
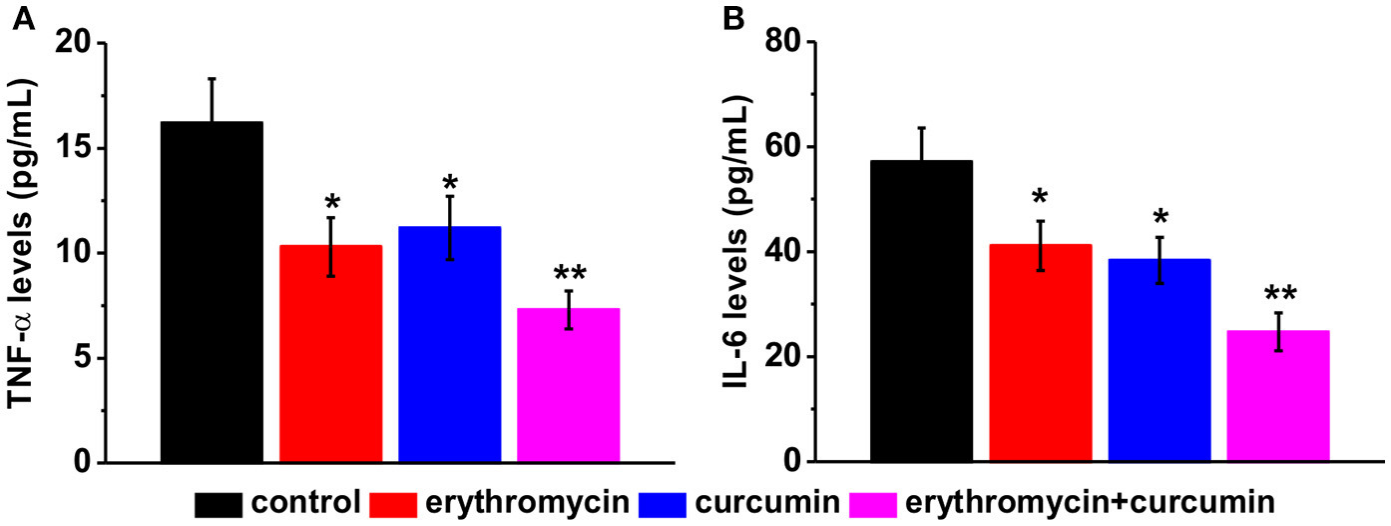
Levels of pro-inflammatory cytokines in the serum of experimental rats after treatment, including TNF-α **(A)** and IL-6 **(B)**, measured by ELISA. Results were presented as mean ± *SD* ^*^p < 0.05 and ^**^p < 0.01 vs. control.

We examined levels of two pro-inflammatory cytokines TNF-α and IL-6 by ELISA. We found that all three treatments (erythromycin monotherapy, curcumin monotherapy, and erythromycin plus curcumin) significantly reduced the levels of TNF-α (Figure 4A) and IL-6 (Figure 4B), with the lowest levels observed in the group of erythromycin plus curcumin treatment. These results suggest that erythromycin and curcumin treatment greatly reduced inflammation induced by osteomyelitis in rats.

## Discussion

Implant-induced osteomyelitis is commonly caused by *S. aureus*. Effective treatment is still lacking due to formation of biofilm making drug difficult to access as well as the generation of antibiotic resistant bacterial strains. Rat models enable us to study new ways to treat osteomyelitis.

In the current study, we successfully established chronic implant-induced osteomyelitis in male Wistar rats as described previously (Lucke et al., 2003; Vergidis et al., 2015; Guzel et al., 2016). Using this model, we studied the effects of erythromycin alone, curcumin alone and erythromycin and curcumin combined treatment against MRSA induced osteomyelitis. We found that although erythromycin or curcumin monotherapy did not show very effective alleviation of bone infection, combined treatment of these two drugs remarkably suppressed bacterial growth, alleviated bone infection status and reduced pro-inflammatory cytokines TNF-α and IL-6. To our best knowledge, this is the first study reporting a significant beneficial effect of erythromycin and curcumin against MRSA infection and osteomyelitis in rats.

Based on its antimicrobial function against *S. aureus*, we originally hypothesized that erythromycin would suppress bacterial growth in our rat model of osteomyelitis that is caused by MRSA infection. Surprisingly, we found that the bacteria levels in the bone gone through 2 weeks of erythromycin treatment did not significantly differ from that of control group. The histopathological status was also similar to control rats. This may be due to resistance of the bacteria toward antibiotics. Minimum inhibitory concentration (MIC) of erythromycin against MRSA in this study was 2.3 mg/L. Thus, MRSA should be resistant to erythromycin according to current literature (Westh et al., 1991). Evolvement of bacteria resistance to antibiotics is a big obstacle for antibiotics in treating *S. aureus* induced infections. This frequently renders antibiotic monotherapy inefficient. In fact, it has been shown previously that addition of erythromycin in cultured mouse osteoblasts at the time of *S. aureus* infection prevented bacteria growth while treatment at 12 h after infection had no such effect (Ellington et al., 2006).

One approach to overcome bacterial resistance is by combining different antibiotics or using herbal medicines together with antibiotics (Allen and Epp, 1978; Hemaiswarya et al., 2008). Some plant-derived products have shown synergistic effect when combining with erythromycin against bacterial infection. For example, organic extracts from Indigofera suffruticosa leaves and erythromycin synergistically suppress *S. aureus* (Bezerra Dos Santos et al., 2015).

Curcumin, a naturally occurring flavonoid, exerts an antibacterial activity against *S. aureus*. It was not clear whether curcumin has any beneficial effect in treating MRSA induced osteomyelitis. In the current study, we investigated this possibility and found only a slight antibacterial effect when treated alone in rats with chronic implant-induced osteomyelitis. Similar to the synergism of curcumin and other antibiotics against *S. aureus*, we also observed a remarkable effect of curcumin and erythromycin combined treatment on suppressing bacteria growth.

Inflammation is associated with bacterial infection. Previous studies showed that increased inflammation is associated with osteomyelitis (Yoshii et al., 2002; Lew and Waldvogel, 2004; Guzel et al., 2016). Despite of its lacking antimicrobial function in MRSA induced osteomyelitis, we did observe a significant reduction of pro-inflammatory cytokines in rats treated with erythromycin alone. This is consistent with previous studies showing that erythromycin can inhibit cytokine gene expressing including TNF-α and IL-6 (Schultz et al., 1998; Cervin, 2001). Curcumin alone or combined with erythromycin also reduced TNF-α and IL-6, with combined treatment showing lower levels of TNF-α and IL-6 compared to curcumin or erythromycin alone. It is worth noting that osteomyelitis model was induced by injection of suspensions of MRSA cell in the current study, which might introduce systemic bacterial infection. Other methods to deliver the MRSA cells locally into the bone, for example the use of agarose beads containing MRSA (Marriott et al., 2005), may reduce the risk of systemic infection. This is one of the limitations of our study.

In addition to anti-microbial and anti-inflammatory efficiencies, development of adverse side effects is a big concern for determination of the ideal treatment. Severe adverse effects could result from antibiotic treatment, including diarrhea, vomiting, malnutrition, and weight loss (Vergidis et al., 2015). We checked our experimental rats closely for side effects resulted from MRSA infection and drug treatment. All the rats tolerated our treatment regimens well, with no rats developing diarrhea or weight loss. In fact, we noticed an increase of body weight when rats were treated with erythromycin or a combination of erythromycin and curcumin. Curcumin treatment alone did not change body weight compared to that of control rats.

Future directions will include determination of potential bacterial resistance, MICs, long term effects, and local application of the drugs. Further studies are needed to determine if combination of erythromycin and curcumin indeed is an ideal therapeutic strategy for treating implant-induced osteomyelitis in human patients.

## Conclusion

We demonstrated here that combination of erythromycin and curcumin has a much stronger antibacterial effect against MRSA in a rat model of implant-induced osteomyelitis than each individual therapy. This treatment was well tolerated, with no death outcome during the experimental period. Our study gives a potential new therapeutic direction for *S. aureus* induced infections and inflammation.

## Author contributions

Did the experiments and analyzed the data: ZZ, CP, YL, YG, WL, PY. Designed the study and wrote the manuscript: XY. All authors approved the final submission.

### Conflict of interest statement

The authors declare that the research was conducted in the absence of any commercial or financial relationships that could be construed as a potential conflict of interest.
